# Hyperautofluorescent Dots are Characteristic in Ceramide Kinase Like-associated Retinal Degeneration

**DOI:** 10.1038/s41598-018-37578-4

**Published:** 2019-01-29

**Authors:** Jesse D. Sengillo, Galaxy Y. Cho, Maarjaliis Paavo, Winston Lee, Eugenia White, Ruben Jauregui, Janet R. Sparrow, Rando Allikmets, Stephen H. Tsang

**Affiliations:** 1 Jonas Children’s Vision Care, and Bernard & Shirlee Brown Glaucoma Laboratory, New York, USA; 2 Department of Ophthalmology, Columbia University, New York, NY USA; 3 Department of Internal Medicine, Reading Hospital, West Reading, PA USA; 4 Frank H. Netter MD School of Medicine, Quinnipiac University, North Haven, CT USA; 5 Department of Internal Medicine, University of Texas Medical Branch at Galveston, Galveston, TX USA; 6 Weill Cornell Medical College, New York, NY USA; 7 Department of Pathology & Cell Biology, Columbia University, New York, NY USA; 8 Institute of Human Nutrition, Vagelos College of Physicians and Surgeons, Columbia University, New York, NY USA

## Abstract

There is a lack of studies which seek to discern disease expression in patients with mutations that alter retinal ceramide metabolism, specifically in the ceramide kinase like (*CERKL)* gene. This cross-sectional case series reports a novel phenotypic manifestation of *CERKL*-associated retinopathy. Four unrelated patients with homozygous *CERKL* mutations underwent a complete ocular exam, spectral-domain optical coherence tomography, short-wavelength fundus autofluorescence (SW-AF), quantitative autofluorescence (qAF), and full-field electroretinogram (ffERG). Decreased visual acuity and early-onset maculopathy were present in all patients. All four patients had extensive hyperautofluorescent foci surrounding an area of central atrophy on SW-AF imaging, which has not been previously characterized. An abnormal spatial distribution of qAF signal was seen in one patient, and abnormally elevated qAF_8_ signal in another patient. FfERG recordings showed markedly attenuated rod and cone response in all patients. We conclude that these patients exhibit several features that, collectively, may warrant screening of *CERKL* as a first candidate: early-onset maculopathy, severe generalized retinal dysfunction, peripheral lacunae, intraretinal pigment migration, and hyperautofluorescent foci on SW-AF.

## Introduction

The ceramide kinase like (*CERKL*, 608381) gene encodes an anti-oxidant protein that, when harboring biallelic mutations, causes an autosomal recessive retinal degeneration^1–7^. The function of CERKL has largely been ascribed to the protection of photoreceptors from oxidative stress, and studies have identified a variety of possible mechanisms, including a role in sphingolipid and ceramide metabolism^1,4,8^ and interactions with calcium sensor proteins in the retina^9^. Recent evidence suggests that CERKL interacts with mitochondrial thioredoxin 2 (TRX2), maintaining TRX2 in a reduced state^10^. TRX2 is critical for maintaining cellular redox balance, and when absent, can lead to hypoxia-induced apoptosis^10–14^. Thus, without properly functioning CERKL, photoreceptors are thought to be more susceptible to oxidative stress and exist in a pro-apoptotic state, leading to severe degeneration of both rod and cone photoreceptors^2,6^.

In one cohort of 272 Spanish retinitis pigmentosa (RP) patients, *CERKL* was found to be a commonly mutated gene, with the p.Arg257Stop mutation being the most frequent^15^. Additional studies have identified missense, splice-site, nonsense, and frameshift mutations in *CERKL* as the cause of retinal degeneration^7,16–18^. However, few studies have sought to characterize the *CERKL*-associated retinopathy phenotype^16–18^. Patients typically exhibit an early maculopathy with severely depressed rod- and cone-responses, which vary in relation to each other. This typical ERG finding and early involvement of the macula have led some to categorize the condition as a cone-rod dystrophy rather than autosomal recessive retinitis pigmentosa (arRP)^18^, as it is traditionally cited^7,15–17^. Other previously described features include lacunae of degeneration in the periphery and intraretinal pigment migration^16–18^.

Early identification and diagnosis are important in the management of patients with *CERKL*-associated retinopathy, as it manifests more severely than most similarly appearing cone-rod and rod-cone dystrophies. This retrospective analysis seeks to further illustrate the *CERKL*-associated retinopathy phenotype, expand on the previously described imaging and electrodiagnostic characteristics, and report a novel SW-AF imaging feature. Four cases of retinopathy caused by homozygous nonsense mutations in *CERKL* are presented. Key features seen on ophthalmoscopy, spectral-domain optical coherence tomography (SD-OCT), en face short-wavelength fundus autofluorescence (SW-AF), quantitative autofluorescence (qAF), and full-field electroretinography (ffERG) are described.

## Materials and Methods

### Subjects

Retrospective review of patient charts and imaging data presented in this study was approved by the Edward S. Harkness Eye Institute and Columbia University Internal Review Boards and adhered to the tenets of the Declaration of Helsinki. The data presented in this study, including images and genetic testing results, are not identifiable to individual patients. Informed consent was obtained as outlined by the Columbia University Medical Center IRB-approved protocol AAAR0284.

### Retinal Imaging

SW-AF and SD-OCT images were acquired by a Spectralis HRA + OCT device (Heidelberg Engineering, Heidelberg, Germany) for all four patients following dilation, as previously described^19^. SW-AF images were acquired using a 30-degree field and 1536 × 1536 pixel resolution with a 486-nm wavelength stimulus and 521 nm barrier filter. An 870 nm light source with real-time registration of an infrared reflectance image was used to acquire all SD-OCT images. Scans were taken horizontally through the fovea (high resolution mode, 9 mm, ART, average of a minimum of 50 images). Quantitative autofluorescence (qAF) was performed and analyzed in patients 2 and 3 (P2 and P3). Protocols for the acquisition of AF images that meet the quality standards necessary for quantification are previously described^20^. Fundus AF images (30°; 488-nm excitation) for these analyses were acquired using a modified Spectralis HRA + OCT camera (Heidelberg, Germany) with the addition of an internal fluorescent reference to correct for variations in laser power and sensitivity (detector gain). Prior to acquisition, the fundus was exposed to the AF light for 20 to 30 seconds to bleach rhodopsin, while at the same time, focus and alignment were refined to produce a maximum and uniform signal over the entire field. Acquired images were analyzed with customized analysis software on the IGOR platform (WaveMetrics, Lake Oswego, OR). The software simultaneously recorded the mean GLs of the internal reference and the area within eight circularly arranged segments positioned at an eccentricity of approximately 7° to 9°–of which the mean value is referred to as qAF_8_. The size of the segments were scaled to the horizontal distance between the fovea and the temporal edge of the optic disc. Control values used in this study consisted of previously published data from 277 healthy subjects (374 eyes; age range, 5–60 years) without a family history of retinal dystrophy^21^.

### Electroretinography

FfERGs (Diagnosys LLC, Lowell, Massachusetts, USA) were recorded in each eye of all patients using either Burian-Allen (BA) contact lenses or DTL recording electrodes in accordance with the International Society for Clinical Electrophysiology of Vision (ISCEV) standards in scotopic and photopic states^22,23^. For two patients (P1 and P2), BA contact lenses were used, and 30 Hz-flicker responses were obtained through narrow band-passed filtering with subsequent computed averaging^24,25^.

### Genetic analyses

For all patients, DNA was isolated from whole blood lymphocytes for whole exome sequencing. Two patient samples (P2 and P4) underwent clinical laboratory improvement amendments (CLIA)-approved whole exome sequencing at the Center of Personal Genomic Medicine (PGM), Columbia University Medical Center (New York, NY). Whole exome sequencing for P1 was performed by the NIHR BioResource (BRIDGE SPEED) study (Cambridge, UK). For P3, whole exome sequencing was performed in the laboratory of Dr. Rando Allikmets at CUMC. The allele frequencies of detected variants in P3 were compared to the Exome Aggregation Consortium (ExAC) (Cambridge, MA; http://exac.broadinstitute.org; accessed March 2018). The possible effect of detected variants was assessed using a combination of prediction programs available through the Alamut software version 2.2 (Interactive Biosoftware, Rouen, France; http://www.interactive-biosoftware.com), using automated computation of this software version.

## Results

### Clinical Data

**Figure 1:**
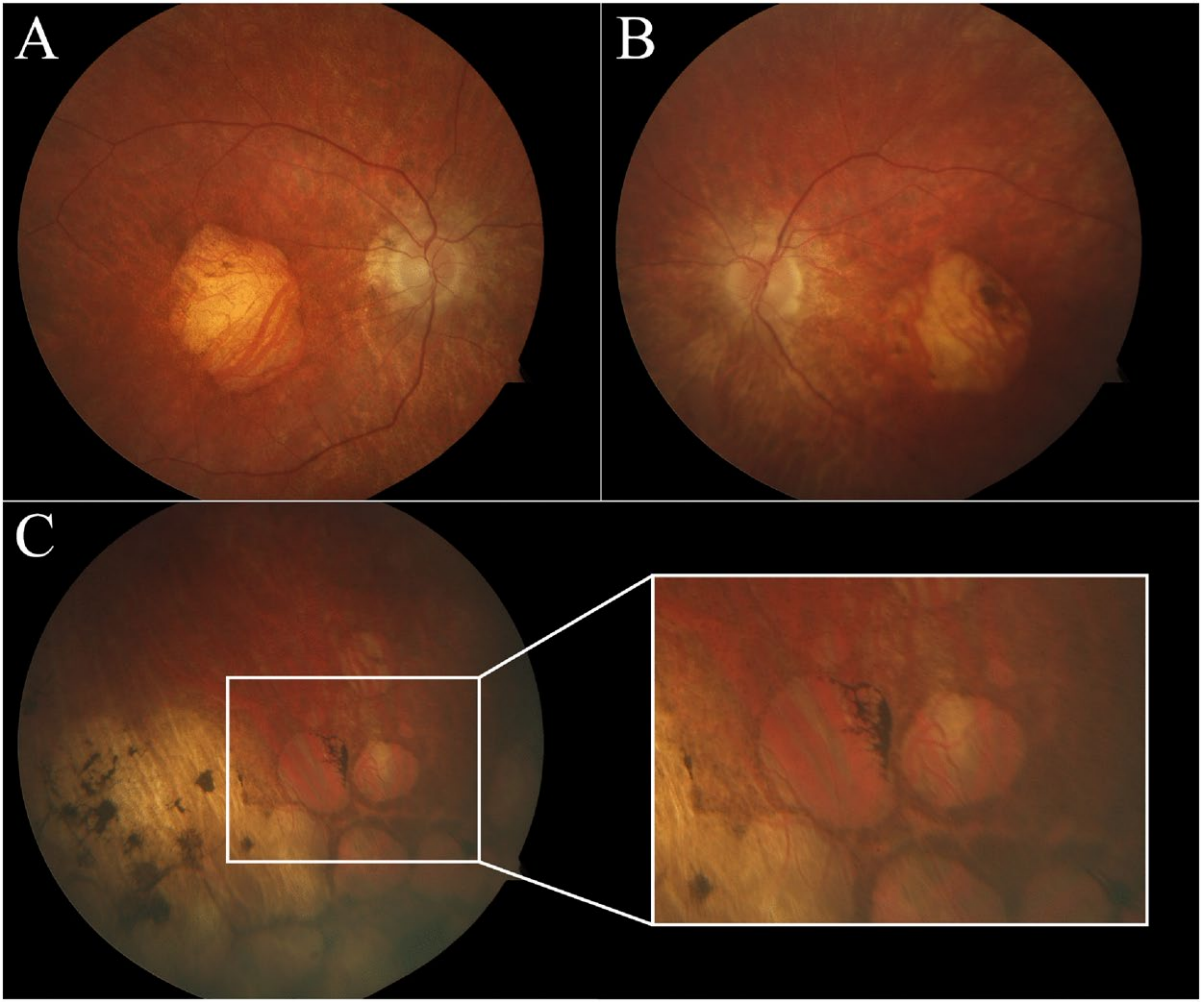
Characteristic fundus in *CERKL*-associated retinopathy. Digital color fundus photographs in the right (**A**) and left (**B**) eyes of P1 with confirmed homozygous frameshift mutations in *CERKL*. An area of macular atrophy is evident, revealing underlying choroidal vessels. A pale disc and attenuated vessels are also seen bilaterally. Peripheral findings (**C**) include punched-out appearing, lacunar-like degeneration with intraretinal pigment migration.

A summary of clinical, demographic and genetic characteristics is presented in Table 1. All patients (mean age, 31.3; range 22–43) reported a family history consistent with autosomal recessive inheritance. Two patients presented with a history of nyctalopia, and all patients described progressive loss of visual acuity. Visual acuity in all patients was not correctable to 20/20, ranging from 20/40 to 20/800 bilaterally (P1) and hand-motion (P4). One patient, P4, exhibited clinically significant posterior subcapsular cataracts in both eyes. All patients presented with extensive maculopathy and retina-wide degeneration on fundoscopy with evidence of chorioretinal lacunar-like degeneration (Fig. 1) in the periphery of two patients (P1 and P4), and bone-spicule pigment deposition in three patients (P1, P2, and P4). Intraretinal pigment observed in P1 appeared to cluster between the peripheral lacunae. A melanocytoma could be appreciated in the left eye nasal to the optic disc of P3.

**Table 1:** Patient Summary. Clinical characteristics of four *CERKL*-associated retinopathy patients.

Patient	P1	P2	P3	P4
Age/Sex	36/M	24/F	22/M	43/F
Ethnicity*	Irish	Irish	Indian	Greek
Genotype	Homozygous p.Arg283Ter	Homozygous p.Arg283Ter	Homozygous p.Arg435Ter	Homozygous p.Arg257Ter
BCVA (OD, OS)	20/800, 20/800	20/50, 20/50	20/50, 20/40	20/HM, 20/HM
Maculopathy	Well-delineated	Bulls-eye	Granular	Well-delineated
Intraretinal Pigment	Yes	Yes	No	Yes
Lacunae	Yes	No	No	Yes
Cataract	No	No	No	PSC**

*Information obtained by history; **PSC; posterior subcapsular cataract.

### Retinal imaging

**Figure 2:**
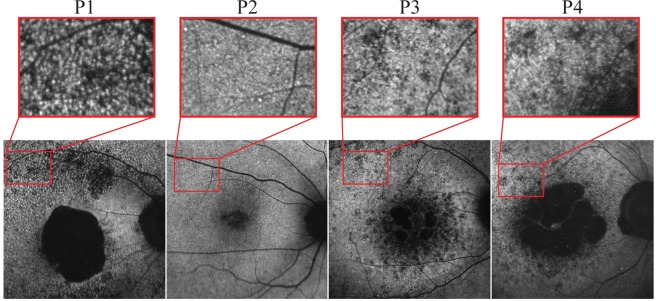
Fundus autofluorescence imaging in *CERKL*-associated retinopathy. SW-AF imaging of four patients with *CERKL*-retinopathy shows large areas of RPE loss in the macula. P1 and P4 show the most well-delineated and largest area of maculopathy. Outside of the area of atrophy in all patients, sparse RPE loss is seen with intermixed numerous hyperautofluorescent foci, generally <120 µm in size.

**Figure 3:**
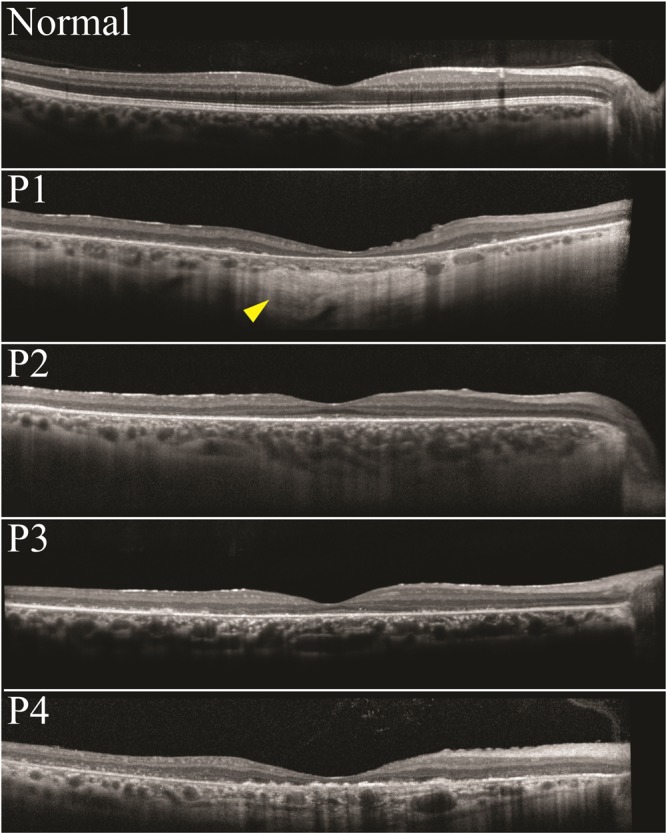
SD-OCT imaging in *CERKL*-associated retinopathy. SD-OCT images are shown for patients P1 through P4. Extensive peripheral thinning and chorioretinal degeneration is seen in all patients. P1 shows the most RPE loss, with increased signal transmittance to the choroidal layers below (yellow arrow).

**Figure 4:**
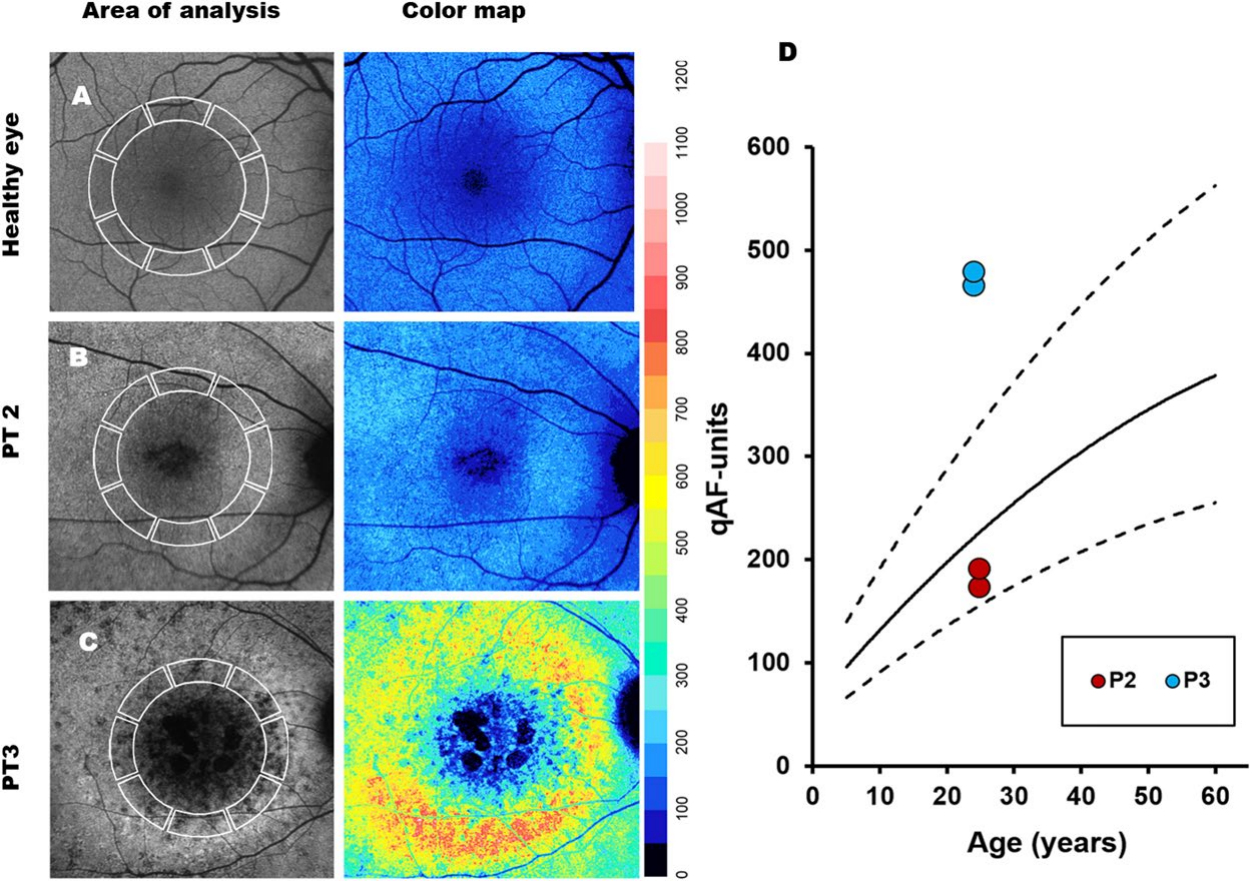
Analysis of quantitative autofluorescence (qAF) in P2 and P3. Average qAF values were calculated in 8 scaled segments within the macula. (**A**) Age and ethnicity-matched healthy individual. (**B**) P2; qAF in P2 were found to be comparable to an age-matched healthy retina but exhibited changes in the spatial distribution of AF (color map). (**C**) P3; qAF in P3 were significantly increased in both eyes with respect to corresponding healthy eyes. (**D**) Plot showing the mean and 95% confidence intervals of qAF in healthy eyes with age and the value of both eyes of P2 and P3.

SW-AF revealed marked atrophy of the central macula in all patients. P1 and P4 had a large, well-delineated area of central atrophy (OD/OS: 9.1mm^2^/9.2mm^2^ and 11.3mm^2^/13.3mm^2^ in P1 and P4, respectively) through which large choroidal vessels were visible on fundoscopy. P4 had some evidence of spared foveal AF signal in both eyes. P2 presented with a more confined bull’s-eye lesion of RPE loss, and P3 exhibited smaller coalescing atrophic lesions. Small hyperautofluorescent foci (generally <120 µm in diameter) of varying confluence were ubiquitous in the areas immediately surrounding the maculopathy and intermittently present among sparsely heterogeneous peripheral RPE atrophy (Fig. 2). An assessment of these foci on SD-OCT was attempted but did not convincingly reveal any spatially corresponding structures. SD-OCT, however, did show extensive atrophy of the outer retinal lamina throughout the entire 30-degree field of view of each patient (Fig. 3). The ellipsoid zone was visibly absent or sparsely granular in all cases. Deterioration of the RPE layer in the macula was most evident on SD-OCT scans in P1, yielding increased signal transmittance to the choroidal layer. No patients exhibited cystoid macular edema. Levels of qAF_8_ in both eyes of P2 fell within the 95% confidence intervals for healthy eyes (Fig. 4D), however, color maps coded according to qAF-units revealed abnormal spatial distribution (Fig. 4B, right column) as compared to an age-matched healthy retina (Fig. 4A, right column). Analyzed qAF_8_ values in P3 in both eyes were significantly increased with respect to corresponding healthy eyes (Fig. 4D) and exhibited >800 qAF-units in regions around the maculopathy (Fig. 4C, center column).

**Figure 5:**
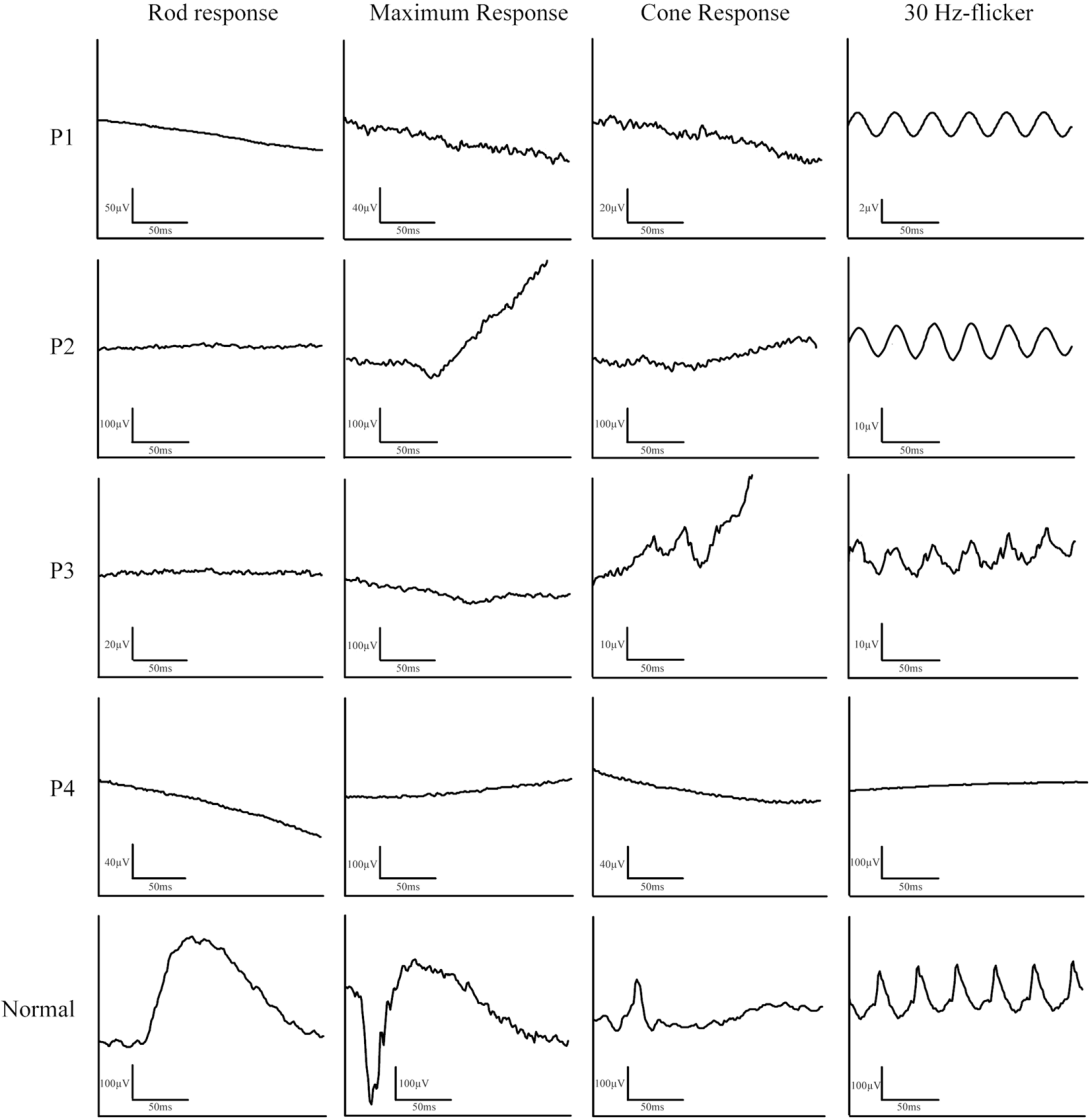
Full-field electroretinography of four patients with homozygous mutations in *CERKL*. FfERG findings show a severe decrease in scotopic and photopic responses in all patients. Note that P1 and P2 underwent 30 Hz-flicker recordings with BA-contact lens electrodes and subsequent narrow band-passed filtering with computed averaging.

Full-field electroretinography (ffERG). All patients presented with marked attenuation of both scotopic and photopic responses, consistent with generalized retinal dysfunction (Fig. 5). Rod-specific b-wave amplitudes were extinguished in all patients. DTL-recording electrodes were used to measure rod and cone function in patients P3 and P4. P3 exhibited 30 Hz-flicker responses less than 10µV in the right eye and 13uV in the left. These responses were non-detectable in P4. A specialized protocol detected residual photopic 30 Hz-flicker responses in two patients (P1, P2), using BA contact lens electrodes, narrow band pass filtering and subsequent computed averaging.

### Genotyping

Disease-causing *CERKL* variants were detected in all patients (Table 1) by whole-exome sequencing (WES). Homozygous c.847 C > T (p.R283*) nonsense variants were identified in P1 and P2, both of Irish descent, and c.769 C > T (p.R257*) was found in P4. The two nonsense variants are predicted to be pathogenic, resulting in the interruption of the reading frame by a premature stop codon in exons 6 and 5, respectively. WES in P3 detected segregating novel homozygous nonsense variants, c.1303 C > T (p.R435*), and compound heterozygous missense variants in *CDH23*, c.429 + 4 G > A (p.?) and c.6197 G > A (p.R2066Q) (AlignGVGD: class 0; SIFT score, 0.36). The allele frequencies of the detected *CDH23* variants were 1.2% and 4.6%, respectively, in the general population of South Asian descent (P3 is Asian Indian), and thus considered frequent, benign variants in individuals from this region.

## Discussion

A dense pattern of hyperautofluorescent foci in regions adjacent to atrophy in the macula was observed for all cases in this study. These foci were least salient in P2 who presented at the earliest disease stage. The discernibility of these foci seems, interestingly, restricted to SW-AF (488-nm excitation) images and exhibit a pattern of accretion with advancing disease severity. These associations and their spatial proximity to degenerative areas may posit a bisretinoid lipofuscin toxicity^26^ pathway in the pathophysiology of *CERKL*-associated retinopathy, although further studies are warranted to investigate this hypothesis. These foci could also theoretically result from accumulated ceramide, as the mutated gene causes metabolic dysfunction in this pathway. We also hypothesize that these foci could be groups of pre-apoptotic photoreceptor cells or RPE as the result of oxidative stress, which CERKL typically reduces. Thinning of the outer nuclear layer was seen on SD-OCT of all patients and intraretinal hyperreflective signals, particularly in P3 and P4, were appreciated within the outer retina. It is possible that this signal represents remnants of degenerated photoreceptors or even debris of RPE cells that have detached from the Bruch’s membrane, which is also seen in other inherited retinal dystrophies. Interestingly, qAF signal in the macula was significantly increased only in regions of advanced disease changes associated with RPE and photoreceptor degeneration (P3) but not in corresponding areas in the macula at an earlier disease stage prior to atrophy (P2). Increased bisretinoid accumulation in photoreceptor outer segments may thus be a downstream event in the pathophysiology of CERKL dysfunction as has been observed in other retinal disease^27,28^.

The phenotype of *CERKL*-associated retinopathy has traditionally been classified clinically under the spectrum of arRP, RP26 (OMIM#608380). Studies in mice interestingly show higher CERKL expression in cones compared to rods^29^, and recent evidence suggest that the clinical presentation and ERG data in three unrelated families are more consistent with a cone-rod dystrophy^18^. Similarly, the presenting symptoms and retinal imaging findings more closely resemble a severe cone-rod dystrophy as opposed to RP in each patient of this case series. Patients in this study experienced early-onset maculopathy with significantly decreased visual acuity at presentation that are atypical findings for most RP cases. Electroretinography is frequently helpful in determining if a dystrophy is representative of a rod-cone (i.e, RP) or cone-rod sequence of degeneration, though this becomes increasingly difficult to determine at later stages of disease. Here, three patients had evidence of residual cone response in the context of non-detectable scotopic B-waves. However, neither a specialized protocol to measure residual rod function nor psychometric testing was performed, yielding any functional comparison between the two cell populations difficult. It would be helpful to assess patients earlier in the disease course to more precisely categorize the electrophysiological phenotype of this condition. The comparatively severe ERG phenotype noted amongst our four patients may also reflect the genotype profile of patients who possess homozygous or compound heterozygous deleterious mutations ranging from nonsense substitutions to single base pair deletions causing a frameshift (i.e., a premature termination in all cases).

Taken together, AF retinal imaging of *CERKL*-associated retinopathy reveals a unique phenotype which may assist specialists in arriving at an accurate diagnosis. For patients with early-onset maculopathy, peripheral lacunae with pigment migration, hyperautofluorescent dots on AF, and severe generalized retinal dysfunction, screening of the *CERKL* gene is suggested as the first, time- and cost-saving, step before whole exome sequencing. WES remains the suggested screening method for patients who exhibit only some of these features. Studies with larger cohorts of *CERKL* patients will be helpful in determining disease progression and further characterizing the ffERG phenotype.

## Data Availability

Datasets generated and/or analyzed during the current study are available from the corresponding author upon reasonable request.
